# Benzimidazolium quaternary ammonium salts: synthesis, single crystal and Hirshfeld surface exploration supported by theoretical analysis

**DOI:** 10.1098/rsos.231094

**Published:** 2024-02-14

**Authors:** Sajid Jamil, Humaira Yasmeen Gondal, Akbar Ali, Ajaz Hussain, Nadia Akram, Muhammad Nisar, Muhammad Nawaz Tahir, Muhammad Ashfaq, Abdul Rauf Raza, Shabbir Muhammad, Zain M. Cheema, Aleena Mustafai, Manal Y. Sameeh

**Affiliations:** ^1^ Institute of Chemistry, University of Sargodha, Sargodha, 40100, Pakistan; ^2^ Department of Chemistry, Government College University Faisalabad, 38000 Faisalabad Pakistan; ^3^ Institute of Chemical Sciences, Bahauddin Zakariya University Multan, 60800, Multan, Pakistan; ^4^ Department of Physics, University of Sargodha, Sargodha, 40100 Pakistan; ^5^ Department of Chemistry, College of Science, King Khalid University, Abha 61413, PO Box 9004, Saudi Arabia; ^6^ Chemistry Department, Faculty of Applied Sciences, Al-Leith University College, Umm Al-Qura University, Makkah 24831, Saudi Arabia

**Keywords:** benzimidazolium, quaternary ammonium salts, synthesis, single crystal and Hirshfeld surface, theoretical analysis

## Abstract

Owing to the broad applications of quaternary ammonium salts (QAS), we present the synthesis of benzimidazolium-based analogues with variation in the alkyl and alkoxy group at N-1 and N-3 positions. All the compounds were characterized by spectroscopic techniques and found stable to air and moisture both in the solid and solution state. Moreover, molecular structures were established through single-crystal X-ray diffraction studies. The crystal packing of the compounds was stabilized by numerous intermolecular interactions explored by Hirshfeld surface analysis. The enrichment ratio was calculated for the pairs of chemical species to acquire the highest propensity to form contacts. Void analysis was carried out to check the mechanical response of the compounds. Furthermore, theoretical investigations were also performed to explore the optoelectronic properties of compounds. Natural population analysis (NPA) has been conducted to evaluate the distribution of charges on the synthesized compounds, whereas high band gaps of the synthesized compounds by frontier molecular orbital (FMO) analysis indicated their stability. Nonlinear optical (NLO) analysis revealed that the synthesized QAS demonstrates significantly improved NLO behaviour than the standard urea.

## 1. Introduction

Quaternary ammonium salts (QAS) comprise a vital class of synthetic products having great significance due to their unique properties like non-flammability, non-volatility, high stability and high polarity [1,2]. They exhibit a wide range of applications in material, medicinal and synthetic chemistry [3]. Benzimidazolium-based QAS receive considerable attention with wide applications [4,5]. These salts having acidic hydrogen at carbon-2 have extensively been used as novel precursors of *N-*heterocyclic carbenes [6] and ionic liquids [7]. In addition, structural similarities with nucleotide make benzimidazolium salts very interesting pharmaceutical scaffold [8]. Based on their substantial applications, many research groups have focused on synthesizing various benzimidazolium salts by different methods such as coupling, cyclization and condensation reactions [9–12]. For instance, Rivas *et al.* synthesized different benzimidazolium salts by palladium [13] and copper-catalysed ring closure [14], whereas Türker *et al*. reported cyanobenzyl substituted benzimidazolium salts by the reaction of *N*-(alkyl)benzimidazole with 3-bromomethylbenzonitrile as effective inhibitors of α-glycosidase (AG), butyrylcholinesterase (BChE) and acetylcholinesterase [14,15]. Benzimidazole ring can also directly convert to benzimidazolium salt by *N*-functionalization [16]. These quaternary ammonium salts demonstrate a range of applications such as ionic liquids [17], fluorescence sensors [18], batteries [19] and solar cells [20]. In addition, these salts have been investigated as enzyme inhibitors by several research groups, and studies explore that these salts acquired inhibitory potential against metabolic enzymes [21].

In the recent time, computational investigation has attracted the scientific community because of their useful applications in order to predict and calculate the optoelectronic properties of the newly prepared organic compound such as peptoids [22], chalcones and *β*-hydroxy carbonyl compounds [23,24], hydrazones [25,26], piperidone derivatives [27], functionalized esters, [28] phosphonates [29], functionalized indoles [30], monocarbonyl curcuminoids [31,32], unsymmetrical acyl thioureas [33], functionalized pyrimidines [34,35], imine-based Zwitterions [36] as well as organic salt systems [37,38]. In continuation of our recent reports on the organic salts [39–43] and benzozole derivatives [44–46], the current study is focused on the design, synthesis and characterization of benzimidazolium salts and the computational exploration of their prospective optoelectronic properties.

## 2. Experimental part

### 2.1. Chemicals and instrumentation

Benzimidazole and alkyl halides were commercially purchased from Sigma-Aldrich. Thin layer chromatography (TLC) was carried out using Merck silica-gel 60F-254 aluminium sheet and visualized under a UV lamp. All compounds were purified by column chromatography using silica gel (100–200 mesh). The ^1^H nuclear magnetic resonance (NMR) spectra were recorded in deuterated methanol (MeOD) using Bruker Advance 400 MHz spectrometer, and ^13^C NMR spectra were recorded at 125 MHz NMR spectrometer in methanol (MeOD). Chemical shift (*δ*) values were determined in parts per million (ppm) while coupling constant (J) in Hertz (Hz). The infrared spectra were recorded on the Perkin Elmer Spectrum RX Fourier Transform–IR System and specific peaks were identified. Bruker Kappa Apex-II CCD diffractometer with APEX-II software was used for data collection. SHELXT-2014 [47] and SHELXL 2019/2 [48] software were used for structure solution and refinement, respectively. H-atoms were placed by using riding model except H-atoms of water solvent in *DBIC, BMBB* and *IMBC*. H-atoms of water were refined freely for getting the correct orientation of them. PLATON [49] software was used for graphical aims.

### 2.2. Chemistry and chemical methods

The N-alkyl benzimidazole (where alkyl = methyl, benzyl [50] was synthesized according to the reported procedures.

### 2.3. General procedure for synthesis of 1-alkylbenzimidazole

The benzimidazole (4 mmol) and 50% aqueous solution of NaOH were added in a round bottom flask. Alkyl halide (5 mmol) was added dropwise and the reaction mixture was stirred for 8 h at room temperature. The reaction was monitored on TLC; after completing, the reaction mixture was extracted with chloroform (30 ml). The solvent was removed under reduced pressure, and the product was purified by column chromatography using ethyl acetate and n-hexane as solvent.

### 2.4. General procedure for synthesis of 1,3-dialkylbenzimidazolium salt (2a-2b)

Alkyl halide (2 mmol) was added into a round bottom flask containing *N-*alkylated benzimidazole (2 mmol) and toluene (25 ml). The reaction mixture was heated under reflux for 18–24 h and monitored by TLC. After the completion of the reaction, the product was filtered and washed with ethyl acetate (15 ml) and the collected precipitates were recrystallized in ethanol to obtained pure crystals.

### 2.5. Synthesis of 3-menthyloxymethyl-1-methyl benzimidazolium chlorides (2c) [51]

Chloromenthyl methyl ether (2 mmol) was added dropwise to 1-methyl benzimidazole (2 mmol) in a round bottom flask under an inert atmosphere. The reaction mixture was allowed to stir for 30 min and diluted with dry diethyl ether. The precipitates were washed with diethyl ether and recrystallized in ethanol to obtain the needle-like crystals of 2c.

#### 2.5.1. 1-benzyl-1-H-benzo[d] imidazole

Solid, yield: 95% IR: 1198.0 (C-N); 1620 (C═N);1646 (C═C aromatic) 2845.7, 2928.6 and 3075.5 (C-H);^1^H-NMR (400 MHz, CDCl_3_): *δ* ppm 8.6 (1H, s). 7.7(2H, d) 7.64 (2H, d), 7.58 (1H, t), 7.34 (3H, t), 7.27 (2H, t), 5.82 (2H, s), ^13^C-NMR, 144.3, 142.8, 138.2, 134.5, 129.6, 127.8, 125.7, 114.3, 51.9.

#### 2.5.2. 1-methyl-1-H-benzo[d]imidazole

Solid, yield: 94% IR: 1220.0 (C-N); 1628 (C═N);1653 (C═C aromatic) 2901.7, 2947.6 and 3003.5 (C-H);^1^H-NMR (400 MHz, CDCl_3_): *δ* ppm 8.9 (1H, s), 7.68 (1H, d), 7.54 (1H, d), 7.44 (1H, t), 7.37 (1H, t), 4.2 (3H, s), ^13^C-NMR, 144.3 137.2, 133.5, 133.5, 126.8, 112.3, 122.8, 36.9.

#### 2.5.3. 1-methyl-3-bytyl-1-H-benzo[d]imidazol-3-ium bromide (2a = BMBB)

Reaction time 24 h (toluene). Yield 91%. Crystalline solid, mp 115–120°C. UV. *λ*_max_ = 276 nm, A = 1.21A IR: 1179.9 (C-N); 1556 (C═N);1962 (C═C aromatic) 2878.5, 2959.2 and 3109.4 (C-H); ^1^H NMR spectrum,(400 MHz, MeOD): *δ* ppm: 8.71 (s,1H), 7.8 (d, 1H), 7.57 (d, 1H), 7.28–7.38 (t, 2H), 5.58 (s, CH_2_), 4.00 (s, CH_3_), 1.77–1.80 (m, CH_2_),1.24–1.28 (m, CH_2_), 0.82–0.84 (t, CH_3_), ^13^C NMR spectrum, ppm: 143.76, 133.33, 131.62, 126.89, 126.84, 129.1, 50.52, 42.47, 14.47, 31.29, 20.71, 13.89.

#### 2.5.4. 1,3-dibenzyl-1-H-benzo[d]imidazol-3-ium chloride (2b = DBIC)

Reaction time 21 h (toluene). Yield 89%. Crystalline solid, mp 204–210°C. UV. *λ*_max_ = 226 nm, A = 1.06A IR: 1189.0 (C-N); 1566 (C═N);1964 (C═C aromatic) 2859.7, 2950.6 and 3070.5 (C-H); ^1^H NMR spectrum,(400 MHz, MeOD): *δ* ppm: 12.1 (2H, s), 7.52 (1H, m), 7.45 (2H, m), 7.4 (4H, m), 7.32 (6H, m), 7.29 (1H, m), 5.96–5.97 (CH_2_, s),5.81–5.82 (CH_2_, s), ^13^C NMR spectrum in ppm: 142.96, 131.12, 131.12, 126.84, 126.84, 114.12, 114.12, 129.1, 128.8, 128.4, 50.06 [52].

#### 2.5.5. 1-((1S,2R,4S)-(+)-menthyloxymethyl)-3-methyl Benzimidazolium chloride (2c = IMBC)

White solid, yield 97%. ${\left[\alpha \right]}_{D}^{25}=+136.50$ (c = 20 mg/5 ml MeOH). *λ*_max_ = 273 nm A = 1.41A IR: *υ* (cm-1) 3389, 2917,(C-H) 1563 (C=N), 1452, 1224(C-N). ^1^H-NMR (400 MHz, chloroform-d): *δ* 12.12 (1H, s), 7.92–7.86 (1H, m), 7.71–7.65 (3H, m), 6.23 (1H, d, J = 12.0 Hz), 5.95 (1H, d, J = 12.0 Hz), 4.28 (3H, s), 3.41 (1H, td, J = 10.6, 4.3 Hz), 2.29–2.22 (1H, m), 1.81–1.71 (1H, m), 1.67–1.58 (1H, m), 1.59–1.43 (2H, m), 1.26–1.17 (1H, m), 0.96–0.77 (3H, m), 0.92 (3H, d), 0.73 (3H, d), 0.14 (3H, d). ^13^C-NMR (100 MHz, chloroform-d): *δ* 144.4, 132.4, 131.1, 127.6, 127.6, 114.5, 112.6, 79.4, 75.4, 48.0, 40.2, 34.3, 33.8, 31.3, 25.4, 22.8, 22.3, 21.0, 15.25 [51].

### 2.6. Computational methodology

All the theoretical calculations were performed using Gaussian 09 [53] at *ω*B97xd/6–31+G(d,p) level [54]. The calculations of frontier molecular orbital (FMO), natural population analysis (NPA) and Nonlinear optical (NLO) analyses were also performed at the same level of DFT. The photophysical properties of compounds 2a–2c were explored by utilizing time-dependent DFT (TD-DFT) method involves range separated functionals (*ω*B97x). The input files were interpreted by Gauss View 5.0 program [55] and the output files were analysed by different software including Avogadro [56], Chem Craft [57], Gauss Sum [58], Multiwfn 3.8 [59] and Origin [60].

### 2.7. Single crystal X-ray diffraction studies of the synthesized benzimidazolium salts 2a–2c

After synthesis, purification and recrystallization, the good-quality crystals were taken for the measurement of single crystal analysis, the experimental details of all these three crystalline compounds are given in table 1.

**Table 1.** Single crystal XRD experimental details of **DBIC, BMBB** and **IMBC**.

compound	**DBIC (2b)**	**BMBB (2a)**	**IMBC (2c)**
CCDC	2279954	2279955	2279956
chemical formula	C_21_H_21_ClN_2_O	C_12_H_19_BrN_2_O	C_19_H_31_ClN_2_O_2_
*M*_r_	352.85	287.19	354.91
crystal system, space group	triclinic, *P*$\bar{1}$	monoclinic, *P*2_1_/*n*	orthorhombic, *P*2_1_2_1_2_1_
temperature (K)	296	296	296
*a*, *b*, *c* (Å)	9.3334 (8), 9.9117 (9), 11.4769 (10)	5.1650 (9), 19.223 (4), 13.864 (3)	6.7238 (5), 8.2781 (8), 36.423 (3)
*α*, *β*, *γ* (°)	68.864 (5), 80.344 (5), 70.019 (5)	94.141 (9)	90, 90, 90
*V* (Å^3^)	929.49 (15)	1372.9 (4)	2027.3 (3)
*Z*	2	4	4
radiation type	Mo *Kα*	Mo *Kα*	Mo *Kα*
µ (mm^−1^)	0.22	2.98	0.20
crystal size (mm)	0.40 × 0.28 × 0.26	0.40 × 0.34 × 0.16	0.38 × 0.32 × 0.24
diffractometer	Bruker Kappa APEXII CCD	Bruker Kappa APEXII CCD	Bruker Kappa APEXII CCD
absorption correction	multi-scan		
(*SADABS*; Bruker, 2007)	multi-scan		
(*SADABS*; Bruker, 2007)	multi-scan		
(*SADABS*; Bruker, 2007)			
no. of measured, independent and			
observed [*I* > 2*σ*(*I*)] reflections	10418, 4144, 2315	11319, 3050, 1365	13864, 3829, 2738
*R*_int_	0.032	0.076	0.048
(sin *θ*/*λ*)_max_ (Å^−1^)	0.658	0.646	0.617
*R*[*F*^2^ > 2*σ*(*F*^2^)], *wR*(*F*^2^), *S*	0.059, 0.191, 1.05	0.060, 0.180, 1.00	0.048, 0.112, 1.01
no. of reflections	4144	3050	3829
no. of parameters	232	191	227
no. of restraints	3	48	3
H-atom treatment	H atoms treated by a mixture of independent and constrained refinement	H atoms treated by a mixture of independent and constrained refinement	H atoms treated by a mixture of independent and constrained refinement
*Δρ*_max_, *Δρ*_min_ (e Å^−3^)	0.42, −0.31	0.54, −0.51	0.13, −0.17

## 3. Results and discussion

### 3.1. Chemistry

**Scheme 1.**
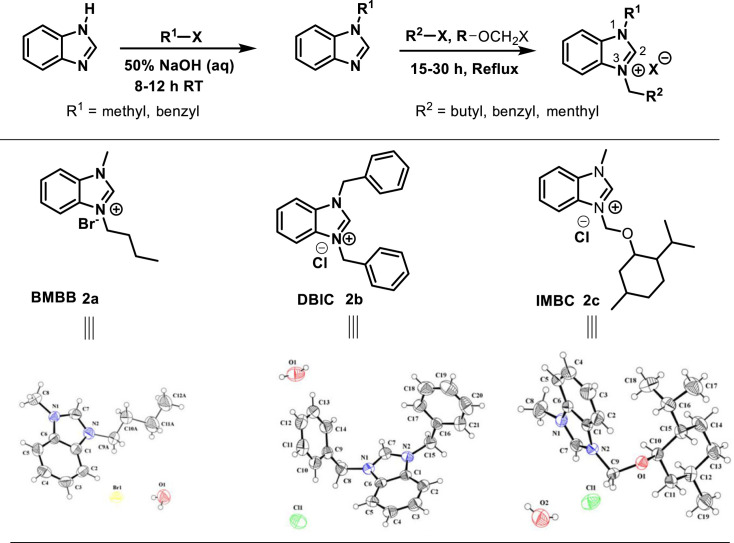
Synthesis of crystalline benzimidazolium quaternary ammonium salts (**2a-c**).

The target benzimidazolium salts were prepared from the *N*-functionalization of benzimidazole in two steps (scheme 1). The first alkylation was carried out under aqueous conditions, where the corresponding alkyl halides were reacted with benzimidazole in the presence of 50% NaOH (aq.) at room temperature. The monoalkylated benzimidazoles were further reacted with butyl, benzyl and menthyloxymethyl halides to obtain dialkylated quaternary benzimidazolium salt refluxing in toluene. The corresponding salts (**2a-c**) were obtained in high yields of 85–95% and further recrystallized in ethanol to recover the pure crystals. Interestingly, all the reactions were carried out under atmospheric conditions where the synthesized quaternary salts were found stable in air and moisture.

### 3.2. Characterization of the benzimidazolium salts 2a–2c

The complete structural elucidation of all purified benzimidazolium salts has been achieved through the comprehensive application of IR, UV, 1H and 13C NMR spectroscopy and ultimately authenticated by single-crystal X-ray diffraction (SC-XRD) analysis. Furthermore, extensive computational studies were employed to gain a deep understanding of the structural and electronic properties of the synthesized quaternary ammonium salts.

#### 3.2.1. Spectral characterization of the benzimidazolium salts 2a–2c

In the ^1^H-NMR spectra, a characteristic singlet appeared in the downfield region at 12.1, 9.1 and 12.1 ppm for **2a-c** respectively, and verifying the resonance of the NCHN protons of the benzimidazolium ring. Notably, for compound **2b**, the H-2 signal was observed at a relatively upfield region (9.1 ppm), which may be attributed to the anisotropic effect of one of the aromatic rings of benzyl groups. The SC-XRD structure of **2a**, presented in scheme 1, demonstrates the orientation of the left-side benzyl ring that the C7 proton resides within the shielding area of the aromatic ring. On the other hand, this effect is absent in compounds **2a** and **2c**, where the H-2 singlet was observed at 12.1 ppm. in the case of compound **2b**, 14 aromatic protons were observed in the aromatic region (7.23–7.98 ppm) due to the presence of two benzyls and a benzimidazole ring. In the H-NMR spectra of compounds **2a** and **2c**, the appearance of singlets for 3Hs at *δ* 5.97 and 5.58 ppm confirmed the presence of (N-CH_3_), whereas the signals appearing in the upfield region indicated the presence of different alkyl groups. A characteristic signal for a diastereotopic methylene (N–CH_2_–O) was appeared at *δ* 6.23 and 5.95 ppm as doublets were observed due to the presence of a chiral centre in the menthyloxymethyl substitution in compound **2c**.

In the ^13^C-NMR spectra of the synthesized compounds **2a-c**, a distinct carbon resonance peak was detected at *δ* 142.4–143.2 ppm, which is attributed to the resonance of C7 carbon of the benzimidazolium ring. Similarly, benzimidazole and benzyl rings (of **2b**) were observed in the aromatic region *δ* 118.6–162.8 ppm, whereas the aliphatic carbons in compounds **2a** and **2c** was verified by a set of peaks appearing in the range of *δ* 53.7–14.2 ppm.

**Figure 1.**
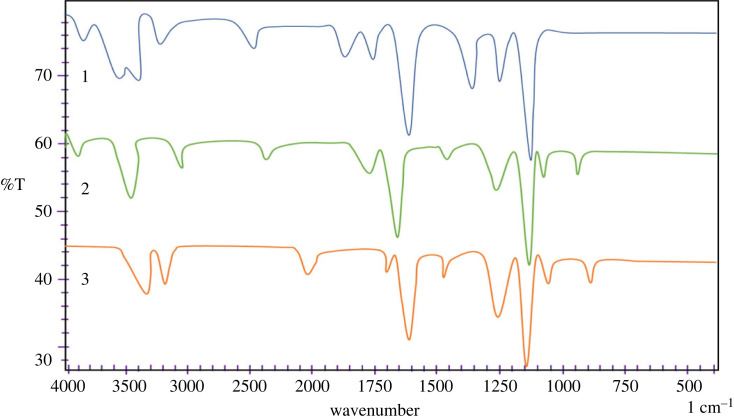
IR spectra of benzimidazolium salts **2a-c**.

IR spectra of compounds **2a–2c** (figure 1) demonstrate the modes of C–N and C–O stretching vibrations at 1100–1250 cm^−1^, whereas intense peaks detected in the range of 1540 to 1590 cm^−1^ were observed for the stretching modes of vibrations of the C=C and C=N in the benzimidazolium salts. Moreover, stretching and vibration absorption above 3360–3440 cm^−1^ were observed for the C-H in both aliphatic and aromatic substitutions.

**Figure 2.**
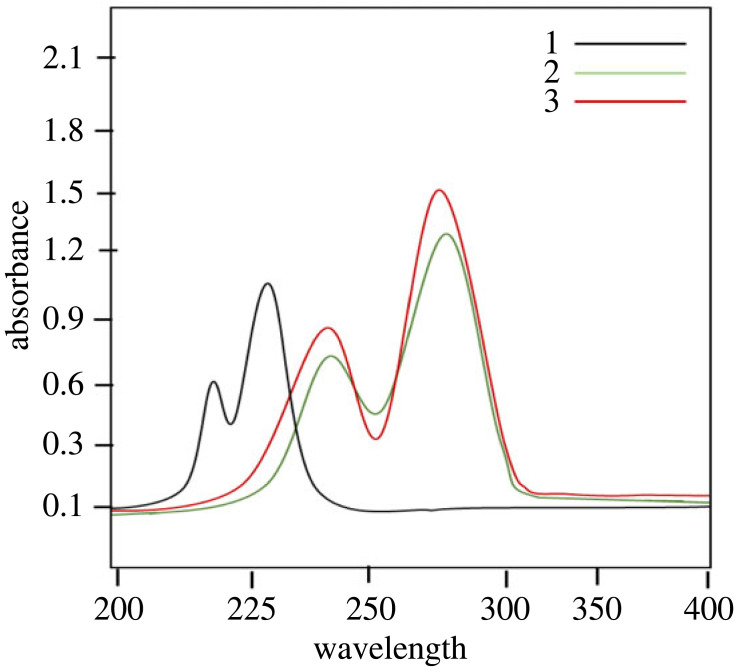
UV-visible spectra of benzimidazolium salts **2a-c**.

The UV-Vis spectra of benzimidazolium salts **2a–2c** in methanol were recorded within 200–400 nm, where the absorption peaks were observed at *λ*_max_ 226, 276 and 273 nm, respectively (figure 2). Compound **2a**, which bears only alkyl substitutions, displayed absorption at a relatively lower wavelength (*λ*_max_ 226) in comparison with **2b**, which features a benzyl group, and **2c**, which includes an alkoxy group.

### 3.3. Single crystal X-ray diffraction studies

**Figure 3.**
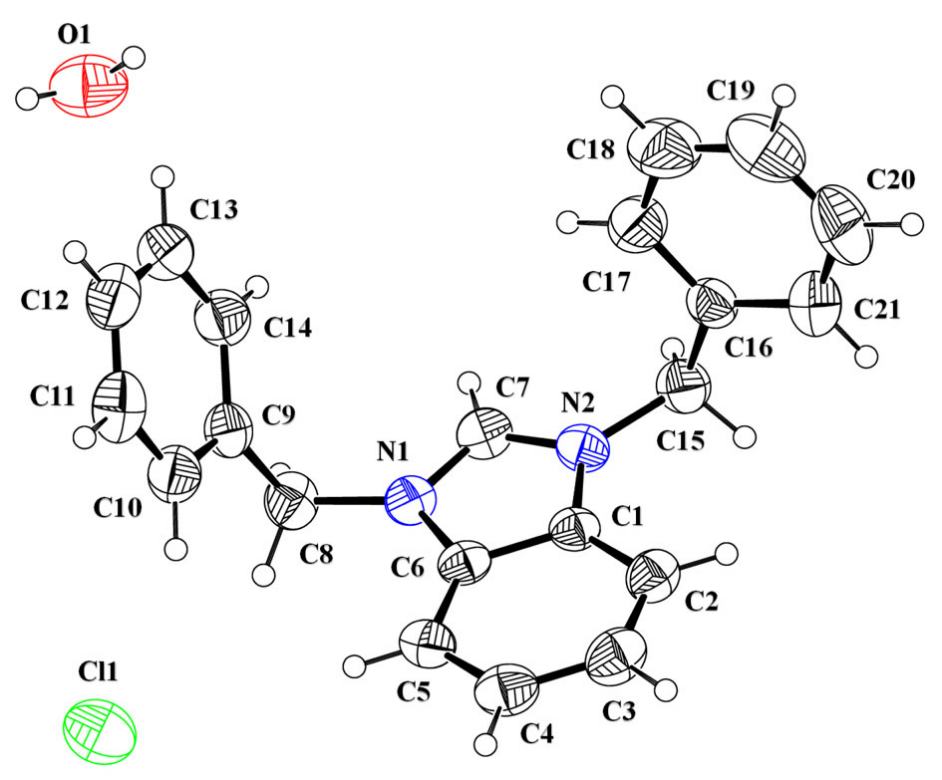
ORTEP diagram of **DBIC** drawn at probability level of 40%. H-atoms are shown by small circles of arbitrary radii.

The asymmetric unit of the salt **DBIC** contained a 1,3-dibenzyl-1H-benzimidazol-3-ium cation (C1-C21/N1/N2), a chloride anion and a water solvent (figure 3, table 1). The central 1H-benzo[d]imidazol-3-ium group A (C1-C7/N1/N2) is planar with root mean square deviation of 0.0052 Å and oriented at the dihedral angle of 73.2 (7)° and 81.8 (7)° with respect to first benzyl group B (C8-C14) and second benzyl group (C15-C21), respectively. The dihedral angles showed that the cation is non-planar.

**Figure 4.**
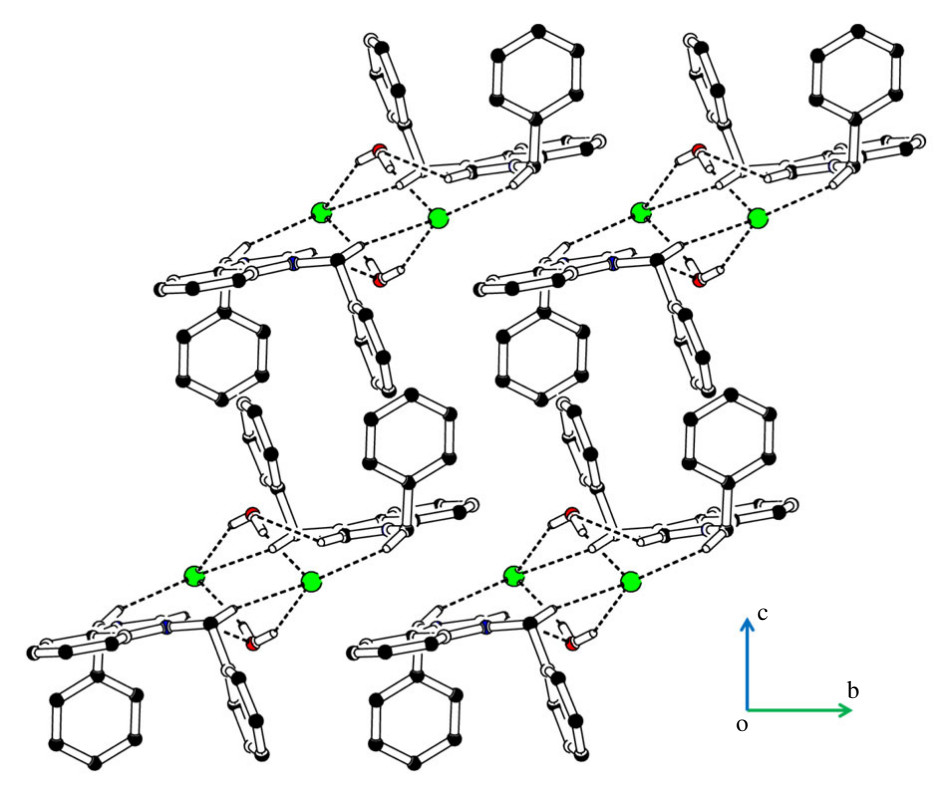
Packing diagram of **DBIC**. Selected H-atoms are shown for clarity.

The cations are not directly interlinked with each other by any H-bonding. Cations are connected with anions by C-H⋯Cl bonding, where CH is from the non-ring CH of benzyl group B and C (figure 4, table 2). No atom of cation acts as H-bond acceptor. The cations are also connected with the water solvent through C-H⋯O bonding, where CH is from the group A. Both H-atoms of water act as H-bond donor for chlorine ions. Each H-atom is involved with H-bonding with a unique symmetry-related chloride anion. So, water solvent plays a significant role in the stabilization of the supramolecular assembly or crystal packing as it forms H-bonding interactions with cation as well as with anion. The water solvent and chloride anions act as bridge to connect cations with each other. The supramolecular assembly is further stabilized by offset π⋯π stacking interactions between rings of the symmetry-related cations with inter-centroid separation range from 3.5231 (14) to 4.2306 (15) Å. Ring offset range is 0.557 to 2.391 Å. Literature crystal structure with CSD [61] reference code JUQWOJ [62] has same cation and a water solvent but has bromide ion instead of chloride ion. EJEMEM [63] has benzyl group attached to one N-atom and methyl group at the other N-atom with bromide ion and water. Bond lengths and bond angles of **DBIC** are consistent with the corresponding ones in related structures. The cation in JUQWOJ and EJEMEM is also not planar as in **DBIC**. The intermolecular interactions in JUQWOJ and EJEMEM are not reported in literature.

**Table 2.** Hydrogen-bond geometry (Å, °) of **DBIC, BMBB** and **IMBC**. Symmetry codes: (i) −*x* + 1, −*y* + 1, −*z* + 1; (ii) *x*, *y*−1, *z* + 1; (iii) −*x* + 1, −*y*, −*z* + 1; (iv) −*x* + 1, −*y* + 1, −*z*; (v) *x*, *y*−1, *z*; (vi) *x* + 1, *y*, *z;* (vii) −*x* + 3/2, *y* + 1/2, −*z* + 1/2; (viii) *x*−1/2, −*y* + 1/2, *z*−1/2; (ix) *x* + 1/2, −*y* + 3/2, −*z;* (x) *x* + 1, *y*−1, *z*; (xi) *x*−1/2, −*y* + 3/2, −*z*.

	*D*—H···*A*	*D*—H	H···*A*	*D*···*A*	*<(D*—H···*A)°*
**DBIC**	O1—H1*A*···Cl1^i^	0.82 (1)	2.45 (2)	3.224 (3)	159 (4)
	O1—H1*B*···Cl1^ii^	0.83 (1)	2.40 (2)	3.204 (3)	164 (4)
	C7—H7···O1^iii^	0.93	2.43	3.244 (4)	146
	C8—H8*A*···Cl1	0.97	2.78	3.668 (3)	153
	C8—H8*B*···Cl1^iv^	0.97	2.72	3.658 (3)	162
	C15—H15*A*···Cl1^v^	0.97	2.66	3.603 (3)	164
**BMBB**	O1—H1*D*···Br1	0.82 (1)	2.56 (1)	3.374 (5)	174 (7)
	O1—H1*E*···Br1^vi^	0.82 (1)	2.53 (2)	3.339 (5)	170 (7)
	C5—H5···O1^vii^	0.93	2.57	3.452 (7)	159
	C7—H7···Br1^viii^	0.93	2.64	3.569 (6)	173
**IMBC**	O2—H2*A*···Cl1^ix^	0.83 (2)	2.40 (2)	3.236 (4)	177 (5)
	O2—H2*B*···Cl1	0.84 (2)	2.35 (3)	3.151 (4)	161 (5)
	C4—H4···Cl1^x^	0.93	2.76	3.519 (4)	139.81
	C8—H8*C*···O2^xi^	0.96	2.37	3.317 (6)	171
	C9—H9*A*···Cl1^vi^	0.97	2.81	3.691 (4)	152

**Figure 5.**
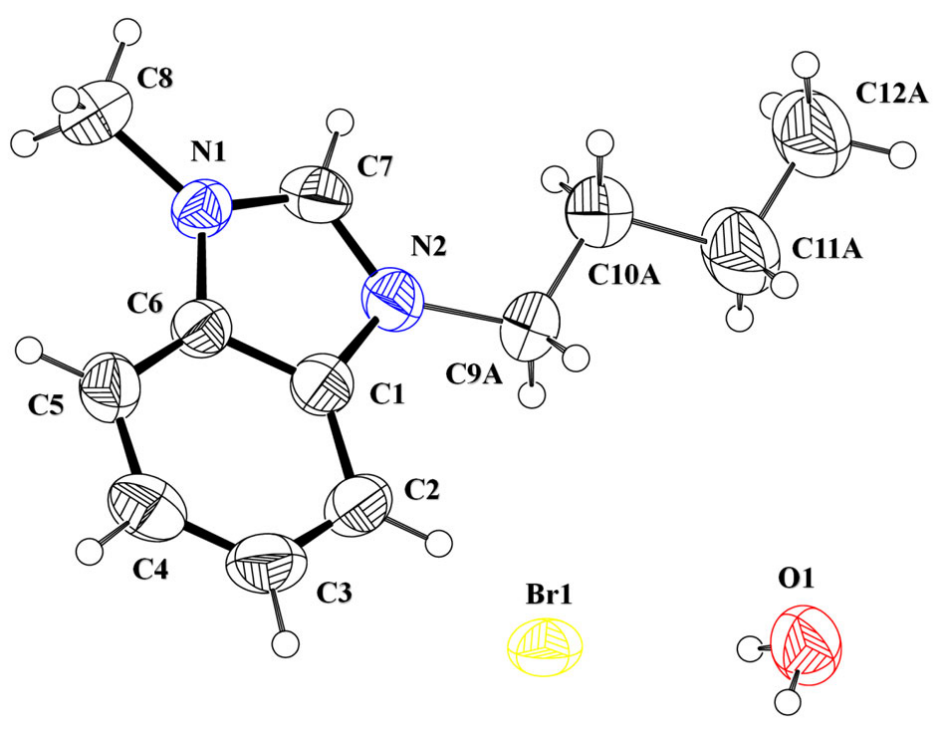
ORTEP diagram of **BMBB** that is drawn at probability level of 40%. H-atoms are shown by small circles of arbitrary radii. Major part of disordered group is shown for clarity.

The asymmetric unit of the salt **BMBB** contained a 3-butyl-1-methyl-1H-benzimidazol-3-ium cation (C1-C8/C9A-C12A/N1/N2), a bromide anion and a water solvent (figure 5, table 1). The central 1H-benzo[d]imidazol-3-ium group A (C1-C7/N1/N2) is bonded with a methyl group and a butyl group B (C9A-C12A). The butyl group is disordered over two sets of sites with occupancy ratio 0.717(13): 0.283(13). The disorder is treated by using various suitable restraints. Group A is planar with root mean square deviation of 0.0138 Å and oriented at the dihedral angle of 15.4 (1)° and 56.5 (1)° with respect to major and minor part of butyl group, respectively. The dihedral angles inferred that the cation is non-planar.

**Figure 6.**
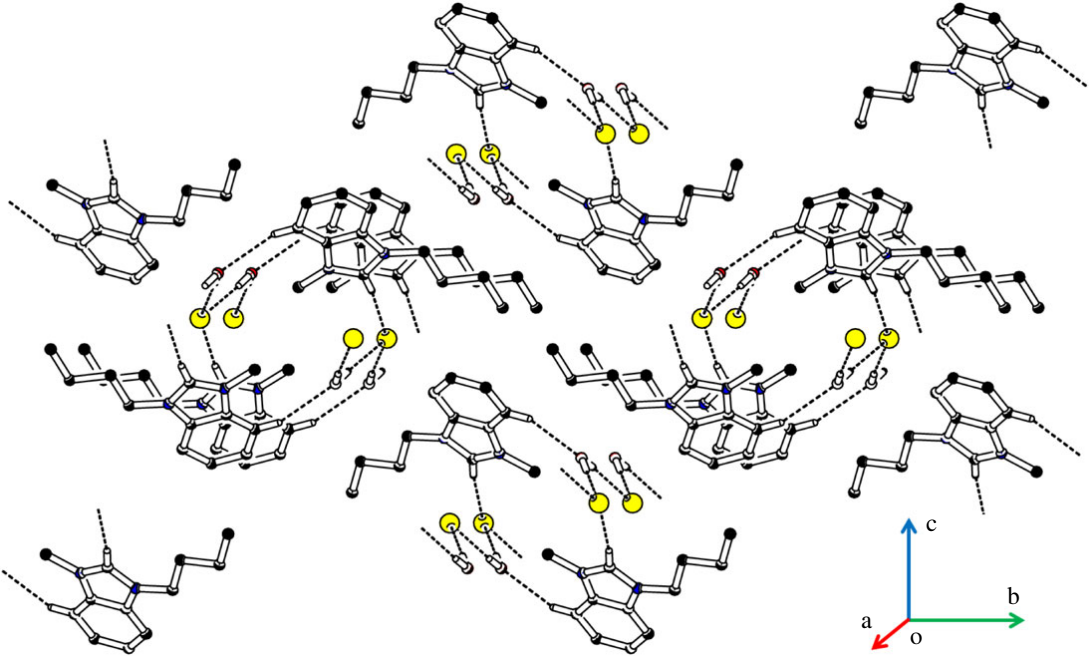
Packing diagram of **BMBB**. Selected H-atoms are shown for clarity.

The cations are not directly interlinked with each other by any H-bonding. Cations are connected with anions by C-H⋯Br bonding, where CH is from group A (figure 6, table 2). No atom of cation acts as H-bond acceptor. The cations are also connected with the water solvent through C-H⋯O bonding, where CH is from the group A. Both H-atoms of water act as H-bond donor for bromide ions. Each H-atom is involved with H-bonding with a unique symmetry-related bromide anion. The water solvent and bromide anions act as bridge to connect cations with each other. The supramolecular assembly is further stabilized by offset π⋯π stacking interactions between rings of the symmetry-related cations with inter-centroid separation of 3.836 (2) Å, and ring offset range is 1.7 to 1.812 Å. Literature crystal structure with CSD reference code KINJUP [64] has isopropyl group bonded with each N-atom of cation and a water solvent but has chloride ion instead of bromide ion, reported as CSD Communication. ACINUY [65] has dinonyl group attached to both N-atoms with bromide ion and water. Bond lengths and bond angles of **BMBB** are consistent with the corresponding ones in related structures. The crystal packing of ACINUY was stabilized by O-H⋯Br bonding, whereas the crystal packing of **BMBB** is stabilized by O-H⋯Br, C-H⋯Br and C-H⋯O bonding.

**Figure 7.**
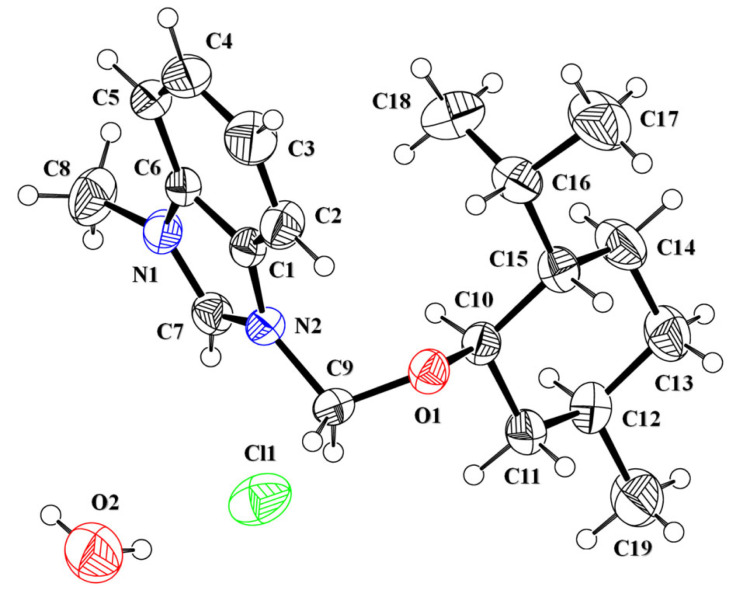
ORTEP diagram of **IMBC** that is drawn at probability level of 40%. H-atoms are shown by small circles of arbitrary radii. Major part of disordered group is shown for clarity.

The asymmetric unit of the salt **IMBC** contained a 3-butyl-1-methyl-1H-benzimidazol-3-ium cation (C1-C19/N1/N2/O1), a chloride anion and a water solvent (figure 7, table 1). The central 1H-benzo[d]imidazol-3-ium group A (C1-C7/N1/N2) is bonded with a methyl group and a 1-isopropyl-2-methoxy-4-methylcyclohexane group C (C9A-C12A). The cyclohexane ring C (C10-C15) is puckered and adopts chair conformation [66]. Group A is planar with root mean square deviation of 0.0105 Å and makes dihedral angle of 88.5 (9)° with group C. The dihedral angle showed that group A is almost perpendicular to group C.

**Figure 8.**
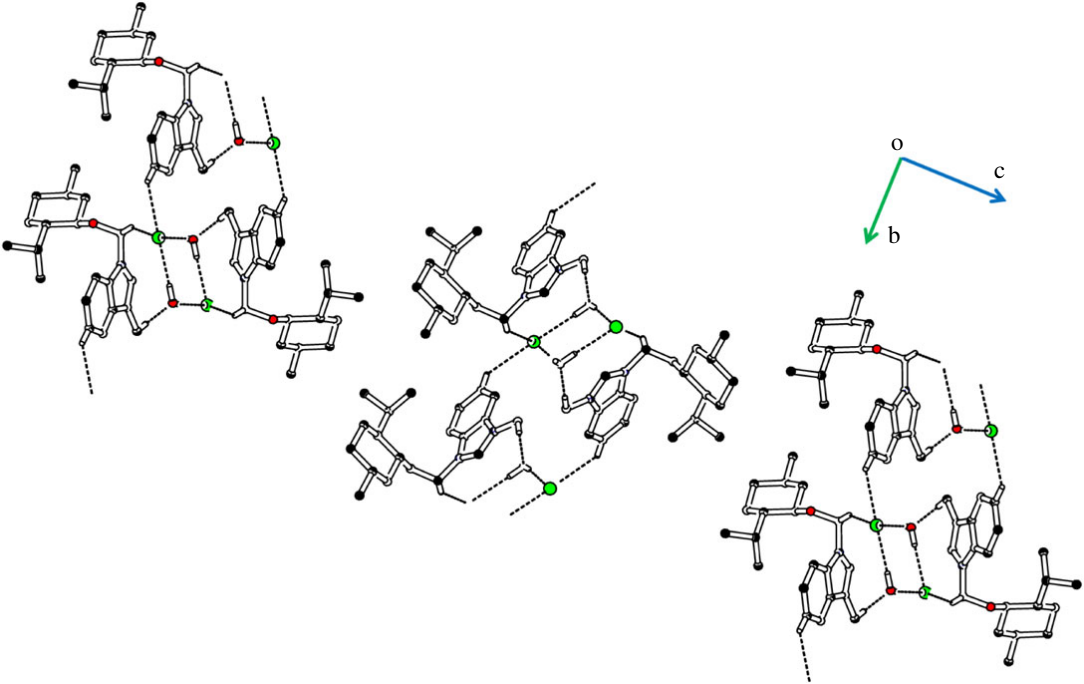
Packing diagram of **IMBC**. Selected H-atoms are shown for clarity.

The cations are not directly interlinked with each other by any H-bonding. Cations are connected with anions by C-H⋯Cl bonding, where CH is from group A and B (figure 8, table 2). No atom of cation acts as H-bond acceptor. The cations are also connected with the water solvent through C-H⋯O bonding, where CH is from the methyl group attached to group A. Both H-atoms of water act as H-bond donor for chloride ions. Each H-atom is involved with H-bonding with a unique symmetry-related chloride anion. The water solvent and chloride anions act as bridge to connect cations with each other. No offset π⋯π stacking interaction of significant strength is found in the crystal packing. Literature crystal structure with CSD reference code ENALEL [67] has cyanobenzyl group bonded with one N-atom and methyl group at the other N-atom of cation, water solvent and a bromide ion. Bond lengths and bond angles of **IMBC** are consistent with the corresponding ones in related structure. The crystal system and space group of **IMBC** is different from the crystal system and space group of ENALEL. ENALEL was crystallized in monoclinic crystal system with space group P2_1_/n (table 3).

**Table 3.** Important bond lengths (Å) and bond angles (°) in **DBIC, BMBB** and **IMBC**.

	**DBIC**	**BMBB**	**IMBC**
N1—C7	1.334 (3)	1.324 (7)	1.318 (4)
N1—C6	1.394 (3)	1.392 (6)	1.384 (4)
N1—C8	1.474 (3)	1.466 (7)	1.465 (5)
N2—C7	1.322 (3)	1.348 (8)	1.333 (4)
N2—C1	1.383 (3)	1.390 (7)	1.393 (4)
C7—N1—C6	107.8 (2)	108.4 (4)	108.9 (3)
C7—N1—C8	124.9 (2)	125.3 (5)	125.7 (3)
C6—N1—C8	127.1 (2)	126.3 (5)	125.4 (3)
C7—N2—C1	108.54 (19)	106.9 (5)	108.1 (3)
N2—C1—C2	131.7 (2)	129.6 (6)	131.9 (3)
N2—C1—C6	106.65 (19)	107.8 (5)	106.3 (3)

### 3.4. Hirshfeld surface analysis

**Figure 9.**
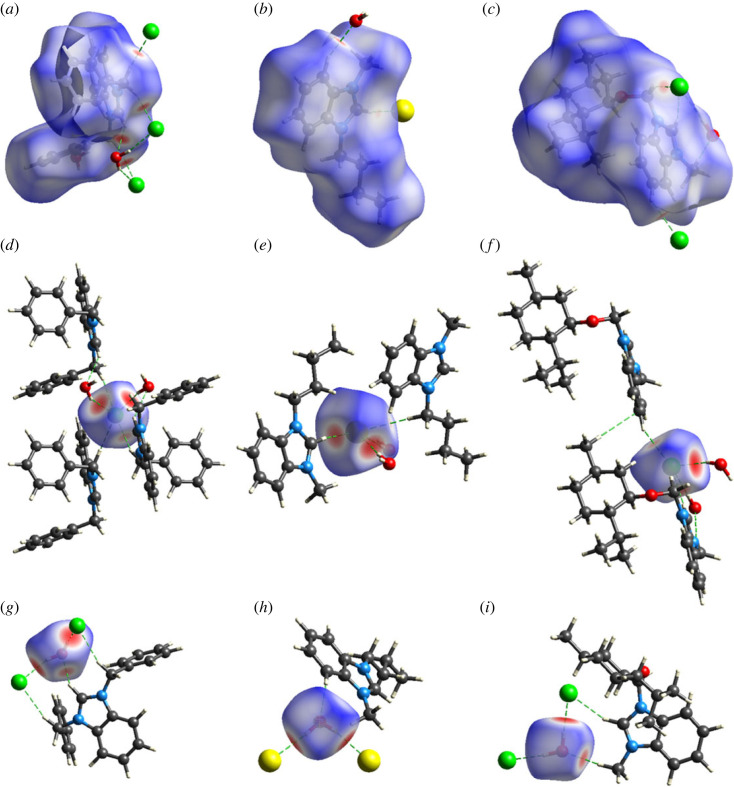
Hirshfeld surface plotted over *d*_norm_ for cation of (*a*) **DBIC**, (*b*) **BMBB**, (*c*) **IMBC**, anion of (*d*) **DBIC**, (*e*) **BMBB**, (*f*) **IMBC**, water solvent of (*g*) **DBIC**, (*h*) **BMBB**, (*i*) **IMBC**.

The interest of researchers in designing new crystals with properties better than the already-known crystals is increasing day by day, and for this, a significant knowledge of the intermolecular interactions is required. In this prospective, we are going to explore the intermolecular interactions in **DBIC**, **BMBB** and **IMBC** by Hirshfeld surface analysis by using Crystal Explorer version 21.5 [68–70]. Hirshfeld surface plotted over normalized distances (*d*_norm_) use colour coding to separate short contacts from the longer ones. Red and blue regions on surface showed short and long contacts, respectively, while the white spots showed contacts with distance equal to sum of VdW radii [71–73]. Figure 9*a–c* are the surfaces for cation of **DBIC**, **BMBB** and **IMBC**, respectively, with red spots showing short contacts. No short contact of significant strength is formed by the cation with the neighbouring cation, but it formed short contacts with anion and water in **DBIC**, **BMBB** and **IMBC**. Short contacts are shown by green dashed lines. Figure 9*d–f* are the surfaces for anion of **DBIC**, **BMBB** and **IMBC**, respectively, with more than one red spot, showing that anion forms more than one short contact. Anion formed short contacts with cation and water. Figure 9*g–i* are the surfaces for water of **DBIC**, **BMBB** and **IMBC**, respectively, with both H-atoms of water and O-atom form short contact. Water does not form any short contact with the neighbouring water molecules.

**Figure 10.**
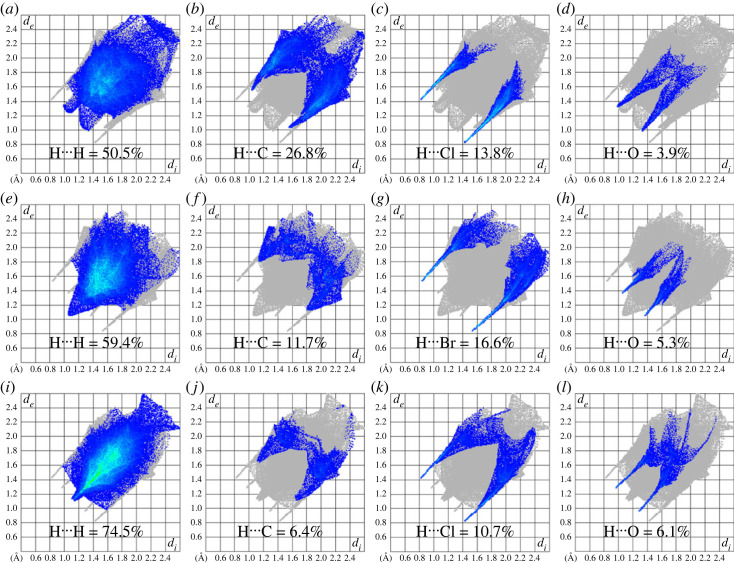
Two-dimensional fingerprint plots for important contacts in (*a-d*) **DBIC**, (*e-h*) **BMBB**, (*i-k*) **IMBC**.

The information that is hidden in the traditional way of crystal packing description can be acquired by two-dimensional finger print plots [74–76]. The plots divide the overall interactions into smaller fragments. Hirshfeld surface containing cation, anion and water is used as input for two-dimensional fingerprint plots analysis. H⋯H contact is the most significant contributor in stabilization of supramolecular assembly in **DBIC**, **BMBB** and **IMBC** with percentage contribution of 50.5% (figure 10*a*), 59.4% (figure 10*e*) and 74.5% (figure 10*i*), respectively. The next larger contributor is H⋯C in **DBIC** whereas H⋯Br and H⋯Cl in **BMBB** and **IMBC**, respectively. Although one water molecule is present in **DBIC**, **BMBB** and **IMBC**, the contribution of H⋯O contact in **IMBC** is larger than the contribution of the corresponding contact in **DBIC** and **BMBB**.

Enrichment ratio provides the probability of the pair of chemical species forming crystal packing interactions with value greater than one for the higher probable contacts [77,78]. Table 4 lists the results of enrichment ratio calculations. H⋯O and H⋯Cl have same enrichment ratio for **DBIC** but these pairs have different enrichment ratio for **BMBB** and **IMBC**. H⋯Cl contact is the most favourable to form crystal packing interactions in **DBIC** and **IMBC** whereas the H⋯O contact is most favourable in **BMBB**. H⋯H contact is slightly favourable in **BMBB** but not favourable in **DBIC** and **IMBC**.

**Table 4.** Enrichment ratio for the pairs of chemical species in **DBIC**, **BMBB**, **IMBC**. Enrichment ratio is not calculated for the pairs with random contact less than 0.99.

contact %	atom	H	C	N	O	Cl/Br
	H	50.5/59.4/74.5	26.8/11.7/6.4	0.5/0.8/1.3/1.3	3.9/5.3/6.1	13.8/16.6/10.7
	C	26.8/11.7/6.4	2.7/1.4/0	1.8/1.6/0	0/0/0.5	0/1.5/0.2
	N	0.5/0.8/1.3	1.8/1.6/0	0/0/0	0/0/0	0/1.7/0.2
	O	3.9/5.3/6.1	0/0/0.5	0/0/0	0/0/0	0/0/0.1
	Cl/Br	13.8/16.6/10.7	0/1.5/0.2	0/1.7/0.2	0/0/0.1	0/0/0
surface%		73/76.6/86.75	17/8.8/3.55	1.15/2.05/0.75	1.95/2.65/3.55	6.9/9.9/5.6
random contacts %						
	H	53.29/58.68/75.26				
	C	24.82/13.48/6.16	2.89/0.77/0.13			
	N	1.68/3.14/1.30	0.39/0.36/0.05	0.01/0.04/0.01		
	O	2.85/4.06/5.81	0.66/0.47/0.24	0.04/0.11/0.05	0.04/0.07/0.11	
	Cl/Br	10.07/15.17/9.72	2.35/1.74/0.40	0.16/0.41/0.08	0.27/0.52/0.38	0.48/0.98/0.31
Enrichment ratio						
	H	0.95/1.01/0.99				
	C	1.08/0.87/1.04	0.93			
	N	0.30/0.2/1.00				
	O	1.37/1.31/1.05				
	Cl/Br	1.37/1.09/1.10	0.00/0.86			

**Figure 11.**
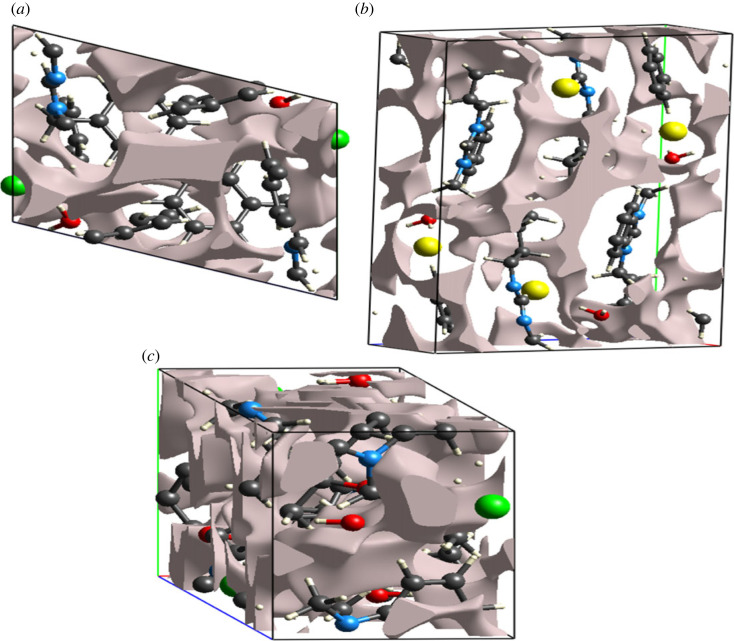
Graphical representation of voids in (*a*) **DBIC**, (*b*) **BMBB** and (*c*) **IMBC**.

The key influencer on the crystal packing and mechanical response of the crystal is the voids and cavities. Poor mechanical response is provided by a crystal with large cavities. Voids are calculated by using an idea of the procrystal electron density. For calculating voids, the isosurface is used by the sum of the atomic electron densities located at appropriate nuclear sites [35,79,80]. Figure 11 is the graphical view of voids in **DBIC**, **BMBB** and **IMBC** mapped on 0.002 au isosurface, where 1 au of electron density = 6.748 e Å^−3^.

The volume of voids in **DBIC**, **BMBB** and **IMBC** is 104.25, 158 and 290.55 Å^3^, respectively. The space consumed by voids in **DBIC**, **BMBB** and **IMBC** is 11.2%, 11.5% and 14.3%, respectively. The voids occupied a small amount of space, which inferred that there is no large cavity, and compounds are expected to have good mechanical response.

### 3.5. Structural properties

**Figure 12.**
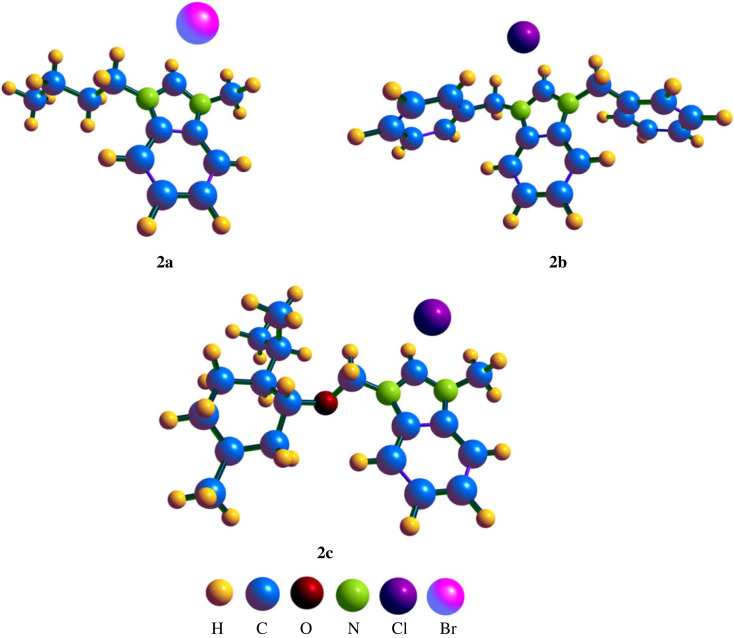
Optimized geometries of compound.

The optimized geometries of the synthesized compounds 2a, 2b and 2c are displayed in figure 12. The optimized geometries of the compounds were calculated at *ω*B97xd/6–31+G(d,p) level of DFT. In compound 2a, the theoretically determined values of bond length N1-C7, N1-C6, N1-C8, N2-C7 and N2-C1 were observed as 1.348, 1.392, 1.450, 1.346 and 1.392 Å, respectively, that were in agreement with the experimentally determined bond lengths (1.324 Å for N1-C7, 1.392 Å for N1-C6, 1.466 Å for N1-C8, 1.348 Å for N2-C7 and 1.390 Å for N2-C1). The values bond angles 107.59°, 123.28°, 128.80°, 107.72°, 131.67° and 106.79° for C7-N1-C6, C7-N1-C8, C6-N1-C8, C7-N2-C1, N2-C1-C2 and N2-C1-C6 respectively were also in accordance with the experimentally determined values of bond angle. Similarly for compound 2b and 2c, the values of bond lengths N1-C7 (1.336 Å for 2b and 1.340 Å for 2c), N1-C6 (1.393 Å for 2b and 1.395 Å for 2c), N1-C8 (1.457 Å for 2b and 1.450 Å for 2c), N2-C7 (1.335 Å for 2b and 1.344 Å for 2c) and N2-C1 (1.397 Å for 2b and 1.395 Å for 2c) were in close approximation with the experimentally determined bond lengths N1-C7 (1.334 Å for 2b and 1.318 Å for 2c), N1-C6 (1.394 Å for 2b and 1.384 Å for 2c), N1-C8 (1.474 Å for 2b and 1.465 Å for 2c), N2-C7 (1.322 Å for 2b and 1.333 Å for 2c) and N2-C1 (1.383 Å for 2b and 1.393 Å for 2c). The values for bond angles for 2b were 107.95°, 123.85°, 125.85°, 108.04°, 132.21° and 106.42° for C7-N1-C6, C7-N1-C8, C6-N1-C8, C7-N2-C1, N2-C1-C2 and N2-C1-C6, respectively. The bond angles in the case of 2c were 107.42°, 123.55°, 125.20°, 107.90°, 131.71° and 106.55° for C7-N1-C6, C7-N1-C8, C6-N1-C8, C7-N2-C1, N2-C1-C2 and N2-C1-C6 respectively. The distance of Br from the carbon of imidazole in 2a was 2.59 Å whereas the distance of Cl with the carbon of imidazole in 2b and 2c was 2.68 and 2.54 Å respectively. Moreover, the coordinates of optimized geometry have been placed in the electronic supplementary material. Furthermore, the structural parameters of the compounds 2a–2c after optimization in gaseous and solvent phase have been placed in electronic supplementary material, table S1 (electronic supplementary material, information).

### 3.6. Computational findings

#### 3.6.1. Frontier molecular orbital analysis

Frontier molecular orbitals consisting of highest occupied molecular orbital (HOMO) and lowest unoccupied molecular orbital (LUMO) play a crucial role in determining the electric, electronic and optical properties of the compounds [51]. In FMO analysis, HOMO–LUMO energy gap (E_LUMO_ - E_HOMO_) value is used to explore the stability and reactivity of the compounds. The small value of energy gap depicts high reactivity and low stability while the large value of energy gap depicts low reactivity and high stability of the compound [81]. The energies of HOMO & LUMO and HOMO–LUMO energy gaps of the compounds are given in table 5. In gas phase, the calculated values of energy gap of 2a, 2b and 2c are 7.757, 7.803 and 8.015 eV, respectively, as presented in table 5. The large values of energy gap indicated the low susceptibility of intramolecular electron transfer which leads to low reactivity of the compounds. The energies of HOMO, LUMO and energy gap for 2a–2c after optimization in solvents of their respective solubilities have also been worked out, which showed a little variation and the data has been provided in electronic supplementary material, table S2 (electronic supplementary material, information).

**Table 5.** Energies of HOMO & LUMO and HOMO–LUMO energy gap.

gas phase			
compounds	E_HOMO_	E_LUMO_	E_g_
2a	−7.381	0.376	7.757
2b	−7.528	0.275	7.803
2c	−7.607	0.408	8.015

**Figure 13.**
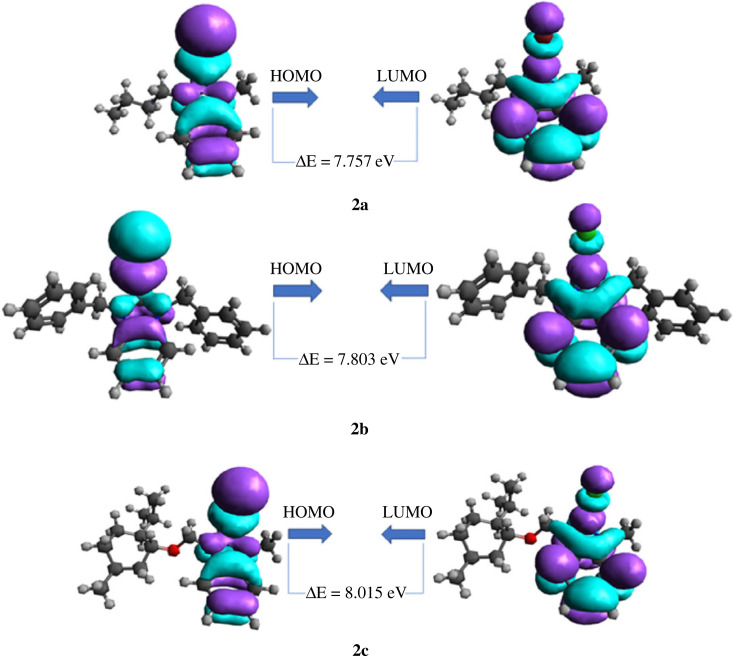
The three-dimensional orbital pictures of HOMO and LUMO of 2a, 2b and 2c.

In compounds 2a, 2b and 2c, the orbital density of HOMO as well as LUMO is scattered over benzimidazole-halogen moiety (figure 13).

#### 3.6.2. Global reactivity parameters

Global reactivity parameter (GRP) descriptors of the compounds can also be calculated by the energies of HOMO and LUMO of the compounds. The GRP descriptors of the compounds including ionization energy (I), electron affinity (A), electronegativity (*X*), global hardness (*η*), chemical potential (*µ*), electrophilicity (*ω*) and global softness (*σ*) can be calculated by the given equations [82],

3.1 $$I=-E_{HOMO},$$

3.2 $$A=-E_{LUMO},$$

3.3 $$\eta =\;\frac{I-A}{2},$$

3.4 $$X=\;\frac{I+A}{2},$$

3.5 $$\mu =\frac{E_{HOMO}+E_{LUMO}}{2},$$

3.6 $$\omega =\frac{\mu^{2}}{2\eta }$$

3.7 $$\mathrm{and}\;\sigma =\frac{1}{2\eta }.$$

The stability and reactivity of the entitled compounds is mainly determined by the values of global hardness (*η*) and global softness (*σ*). The value of global hardness is maximum in the case of 2c (4.007) that illustrated the high stability and low reactivity of 2c, whereas the value of global hardness is smallest for 2a (3.878) that illustrated the low stability and high reactivity of 2a. Similarly, the greater magnitude of global softness of 2a (0.128) depicts the low stability and high reactivity of 2a whereas small value of global softness of 2c (0.124) depicted its greater stability and lowest reactivity. The values of GRP descriptors of the compounds are given in table 6.

**Table 6.** GRP descriptors of compounds 2a–2c.

GRP descriptors	ionization energy (*I*)	electron affinity (*A*)	electronegativity (*Χ*)	global hardness (*η*)	chemical potential (*µ*)	electrophilicity (*ω*)	global softness (*σ*)
**2a**	7.381	−0.376	3.502	3.878	−3.502	1.581	0.128
**2b**	7.528	**−**0.275	3.626	3.901	−3.626	1.685	0.129
**2c**	7.607	−0.408	3.599	4.007	**−**3.599	1.616	0.124

#### 3.6.3. Density of states analysis:

**Figure 14.**
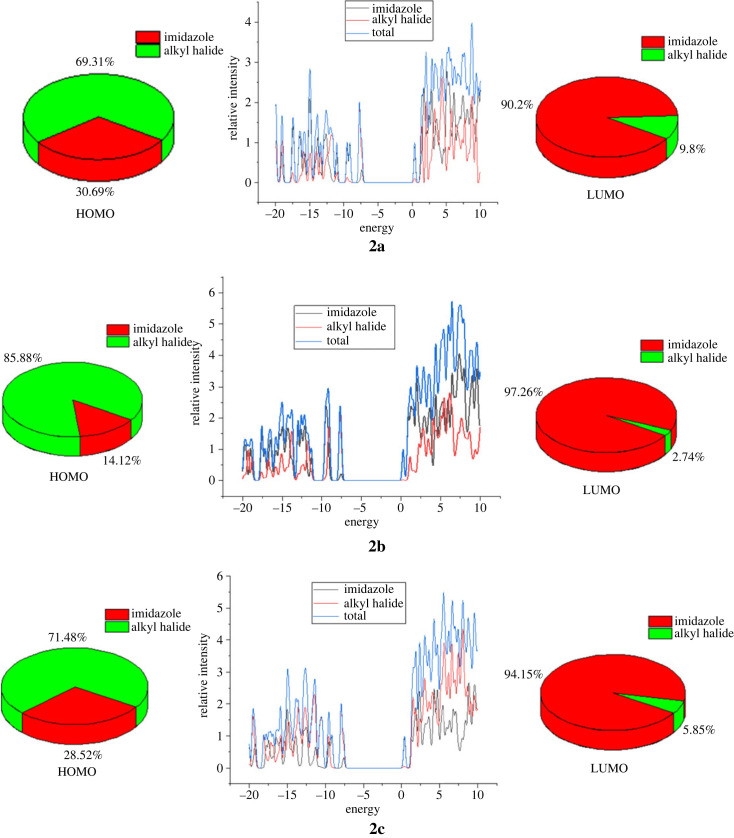
DOS spectra and percentage contribution of fragments of 2a–2c.

Density of states (DOS) analysis describes the frequencies of electronic excitation that occur per unit of energy and volume. The understanding of likelihood of possible states per unit volume and energy is necessary to assess a wide range of electronic activities such as total electron scattering, excitation and *λ*_max_ of the novel materials. The dispersion of electrical characteristics between HOMOs and LUMOs alters as a result of the presence of various electron-activating and deactivating groups in the compounds. The positive values along the x-axis reflected the electrical configuration of LUMOs, while the negative values represented the conductive channel at HOMO (figure 14). The difference that prevailed between these values represents respective energy deficit. In compound 2a, 30.69% of HOMO composition was contributed by imidazole, and 69.31% of HOMO composition was contributed by alkyl halide, whereas 90.02% of LUMO was contributed by imidazole, and 9.8% of LUMO was contributed by alkyl halide. Similarly, in the case of compound 2b, 14.12% and 85.88% HOMO was contributed by imidazole and alkyl halide, while 97.26% and 2.74% of LUMO was contributed by imidazole and alkyl halide, respectively. The 28.52% and 71.48% of HOMO of compound 2c was contributed by imidazole and alkyl halide moiety, respectively, whereas 94.15% and 5.85% of LUMO was contributed by imidazole and alkyl halide, respectively.

#### 3.6.4. Nonlinear optical analysis

The study of optical properties of compounds contributes a lot in the domain of optoelectronics [83]. NLO effect is the result of the interaction of electromagnetic field from several media with the prepared compounds to produce a new field with distinct propagation properties including amplitude, phase and frequency [84]. The materials showing notable NLO effect have significant applications in the field of optical switching, digital signal processing and communication technology [51]. The NLO properties of the respective compounds can be illustrated by comparing their effects with those of urea showing the hyperpolarizability of 0.23 × 10^−23^ e.s.u and dipole moment of 1.3732 Debye (D) [85]. The NLO properties such as dipole moment (*μ*), polarizability (*α_total_*) and hyperpolarizability (*β*_total_) of the compounds can be determined by the equations (3.8), (3.9) and (3.10), respectively.

3.8 $$\mathrm{Dipole}\;\mathrm{moment}(\mu )={\left(\mu_{x}^{2}+\mu_{y}^{2}+\mu_{z}^{2}\right)}^{1/2},$$

3.9 $$\mathrm{Polarizability}(a_{\mathrm{total}})=\frac{\left(a_{xx}+a_{yy}+a_{zz}\right)}{3},$$

3.10 $$\begin{array}{rl} \mathrm{Hyperpolarizability}(\beta_{\mathrm{total}})= & [{\left(\beta_{xxx}+\beta_{xyy}+\beta_{xzz}\right)}^{2}\\ +{\left(\beta_{yyy}+\beta_{xxy}+\beta_{yzz}\right)}^{2}\\ {+{\left(\beta_{zzz}+\beta_{xxz}+\beta_{yyz}\right)}^{2}]}^{1/2}. \end{array}$$

The calculated values of *μ* for 2a, 2b and 2c were found to be 7.940, 9.041 and 7.069 D, respectively (table 7). The values of *μ* for all the prepared compounds were greater than that of urea (which shows *μ* value of 1.3732 D), contributing towards relatively better hyperpolarizability of the compounds. Furthermore, the magnitudes of linear polarizability (*α_t_*_otal_) of compounds 2a, 2b and 2c were equal to 185.801, 269.453 and 255.931 arb. units respectively (mentioned in table 8) and the values of hyperpolarizability (*β*_total_) for compounds 2a, 2b and 2c were 587.001, 918.212 and 661.606 arb. units, respectively (given in table 9), greater than that of urea (reported hyperpolarizability of 0.23 × 10^−23^ e.s.u and 44.1 arb. units calculated from DFT) reflecting that expected NLO response of prepared compounds might be better than urea. The values of *μ*, *α*_total_ and *β*_total_ of compounds 2a–2c, achieved after their optimization in solvent phase have also been displayed in tables 7–9.

**Table 7.** Dipole moment of compounds 2a–2c.

dipole moment (Debye)	gas phase			solvent		
	2a	2b	2c	2a	2b	2c
*μ_x_*	−2.9677	−2.7180	1.4587	−6.8625	4.3436	4.5624
*μ_y_*	6.0136	−7.7228	4.2761	8.7198	−14.8221	−14.8880
*μ_z_*	4.2520	3.8374	−5.4375	5.3111	0.6374	−3.4333
*μ_total_*	7.9404	9.0418	7.0696	12.3019	15.4586	15.9454

**Table 8.** Linear polarizability of compounds 2a–2c.

linear polarizability	gas phase			solvent		
	2a	2b	2c	2a	2b	2c
*α_xx_*	194.928	331.844	283.921	194.781	324.362	262.213
*α_yy_*	218.464	254.292	290.049	210.888	258.174	272.722
*α_zz_*	144.013	222.224	193.823	153.199	220.831	210.933
*α_total_*_(arb. units)_	185.801	269.453	255.931	186.289	267.789	248.622

**Table 9.** Hyperpolarizability of compounds 2a–2c.

hyperpolarizability	gas phase			solvent		
	2a	2b	2c	2a	2b	2c
*β_xxx_*	110.816	−11.507	94.250	−147.406	165.077	−20.201
*β_xxy_*	−74.366	−1.008	−52.247	321.993	−222.403	−176.370
*β_xyy_*	−129.742	281.509	−28.824	−652.244	107.961	189.388
*β_yyy_*	596.766	743.956	346.793	1106.463	−835.660	−820.836
*β_xxz_*	−31.950	76.946	92.781	133.297	8.777	−84.598
*β_yyz_*	211.284	229.209	−275.472	399.549	35.452	−157.584
*β_xzz_*	6.023	4.145	−1.342	−47.715	24.587	21.370
*β_yzz_*	54.194	61.323	267.646	110.917	−128.818	−191.525
*β_zzz_*	−70.049	41.848	−160.174	65.331	−11.051	−143.134
*β*_total(arb. units)_	587.001	918.212	661.606	1856.209	1224.078	1264.065

#### 3.6.5. UV-visible spectroscopic analysis

**Figure 15.**
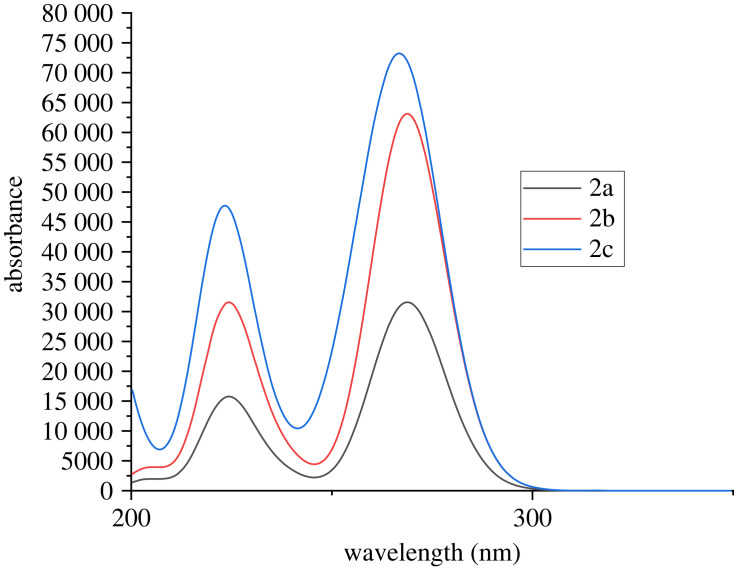
Graphical representation of UV-Vis analysis of compounds 2a–2c.

The theoretical calculations of UV-Vis spectral data of compounds 2a–2c were carried out by applying TD-DFT which allows the evaluation of medium sized molecules [86]. The UV-vis spectroscopic analysis was also performed at *ω*B97xd/6–31+G(d,p) functional level in the presence of methanol as solvent. Theoretically obtained values of energy, wavelength, oscillator strength and major contribution of molecular orbitals are mentioned in electronic supplementary material, table S3 (electronic supplementary material, data). The calculated values of *λ*_max_ for compound 2a, 2b and 2c in methanol were found to be 268.457, 268.457 and 256.308 nm, respectively. The UV-Vis spectrum of the compounds is shown in figure 15.

#### 3.6.6. Natural population analysis

**Figure 16.**
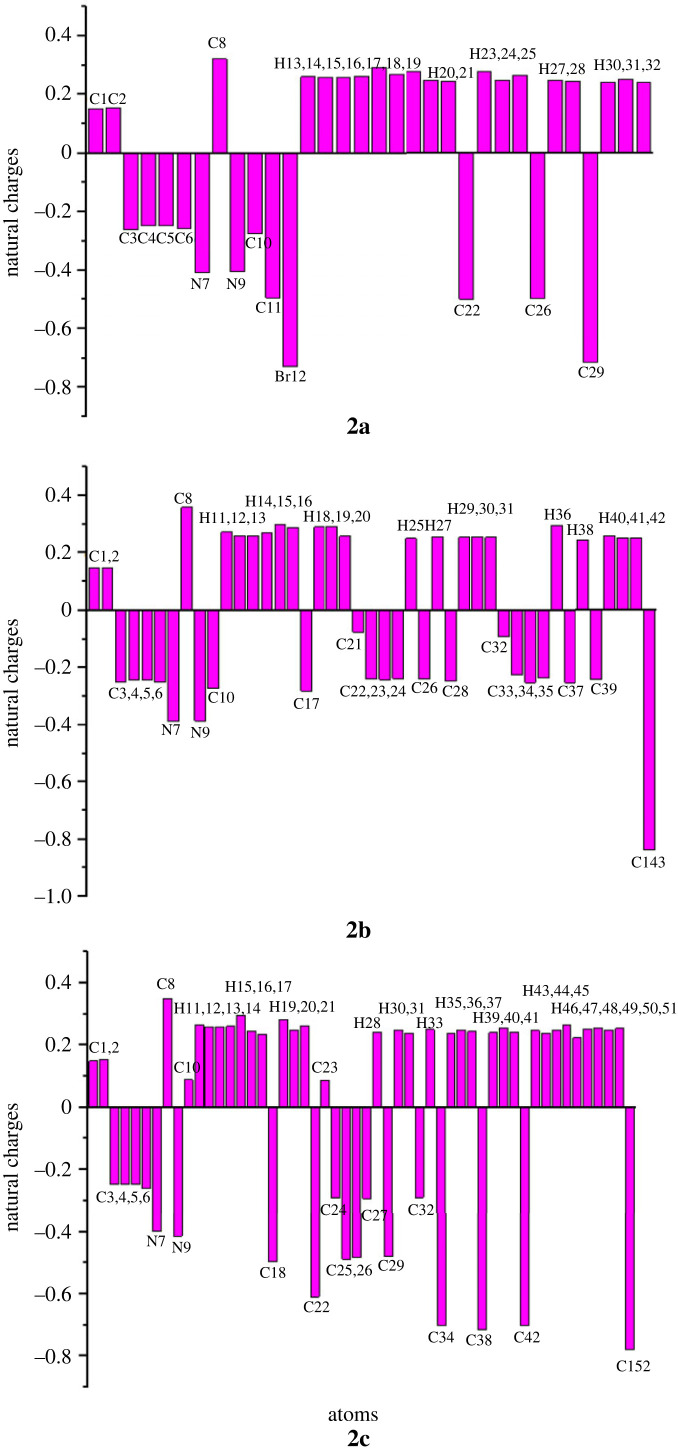
NPA of compounds 2a–2c.

NPA was carried out to determine the charge on each atom of the entitled compounds. The calculations of NPA were performed using Gaussian 09 software by applying *ω*B97xd/6–31+G(d,p) functional level. In the case of 2a, the largest value of negative charge −0.727 e^−^ was present over bromine (Br12). In 2c, the greater value of negative charge −0.779 e^−^ was observed over chlorine (Cl52). Similarly, the greater negative charge −0.838 e^−^ in 2b was also present over chlorine (Cl43). However, in all the three compounds the maximum positive charge was exhibited by carbon number 8 (C8) due to its direct bond with two highly electronegative nitrogen atoms. The magnitude of positive charge on C8 of 2a, 2b and 2c was 0.321 e^−^, 0.359 e^−^ and 0.349 e^−^respectively. The graphical representation of NPA of the compounds is shown in figure 16.

#### 3.6.7. Molecular electrostatic potential analysis

Molecular electrostatic potential (MEP) analysis is frequently employed for the visual examination of charge distribution in the molecules of interest. MEP plots are used to locate the regions of nucleophilic and electrophilic attack on the molecule [87]. The MEP (V(r)) map around a respective molecule at a given point r along axis is interpreted by the interaction energy between proton present at point r and electrical charge generated by the electrons and nuclei of the molecule. MEP maps can also be defined by the following equation [88]:

3.11 $$V(r)=\sum \left(\frac{Z_{A}}{R_{A}}-r\right)-\frac{\int^{\;p(r^{\prime })}}{\left(r^{\prime }-r\right)}dr^{\prime }.$$

**Figure 17.**
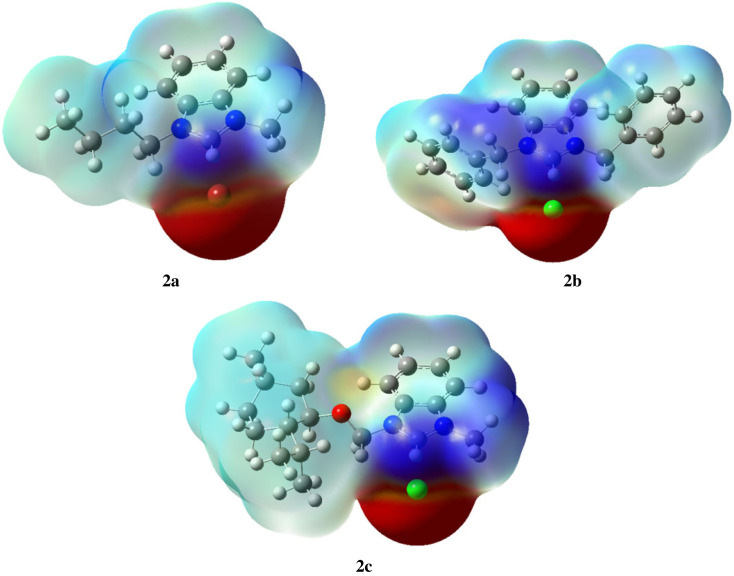
MEP plots of compounds 2a–2c.

The MEP maps of the prepared compounds **2a–2c** (given in figure 17) consist of various coloured tones (ranging from green to red) revealing different electrostatic potential of the prepared compounds. The red colour area stipulates electrophilic reactive site whereas blue colour stipulates nucleophilic reactive site of the molecules. In all the three compounds 2a–2c, red colour area around halogens (Br and Cl) encompasses their negative electrostatic potential, whereas the blue colour area over the C-H of imidazole encompasses their positive electrostatic potential.

#### 3.6.8. Electron localization function and localized orbital locator analysis

**Figure 18.**
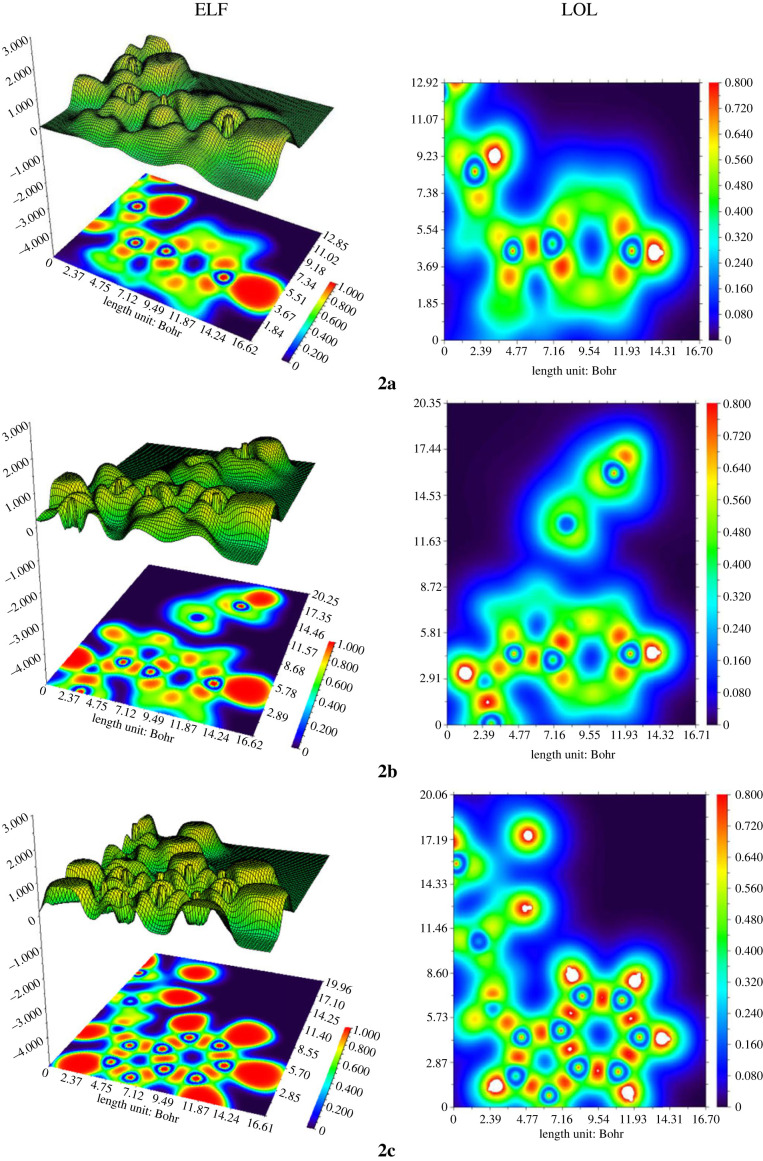
ELF and LOL analysis of compounds 2a–2c.

Electron localization function (ELF) and localized orbital locator (LOL) analysis are useful techniques to determine the electron density concentration in the entitled compounds. Through covalent bond analysis, ELF and LOL identify primary active sites in the molecules [89]. The coloured projection charts and contour maps of ELF and LOL (displayed in figure 18) for the prepared compounds were derived using Multiwfn program. The ELF maps were designed in the range of 0 to 1.0 denoting their analogous chemical content. The value of ELF in the range of 0.5 to 1.0 represents bonding and non-bonding localized electrons while ELF magnitude less than 0.5 represent delocalized electrons. LOL expresses more conclusive and flawless representation of electron density concentration in the entitled compounds by the study of topological parameters such as kinetic energy density. Furthermore, the red and blue colours in the scale represent high and low ELF and LOL values.

#### 3.6.9. Fourier transform–infrared analysis

DFT calculations were executed to determine the vibrational frequencies of compound 2a–2c. The FT-IR results obtained by DFT analysis were compared with experimental data as given in electronic supplementary material, tables S4–S6. Moreover, the simulated IR spectra for compounds 2a–2c have been placed in electronic supplementary material, figures S1–S3, respectively.

##### 3.6.9.1. C=C vibration

The IR bands for C=C stretching was observed at 1549.38 cm^−1^ for compound 2a and at 1550.80 cm^−1^ for compound 2c. The C=C stretching frequencies for three benzene rings of compound 2b were found to be 1543.83 cm^−1^ for Ben1, 1507.79 cm^−1^ for Ben2 and 1511.57 cm^−1^ for Ben3. The experimentally determined values for C=C vibrations in compound 2a, 2b and 2c were 1962, 1964 and 2218 cm^−1^, respectively.

##### 3.6.9.2. C=N vibration

The C=N stretching frequencies in the imidazole rings of compound 2a, compound 2b and compound 2c were found to be 1605.60, 1625.48 and 1621.20 cm^−1^, respectively. The experimental values for C=N vibration 1556 cm^−1^ (2a), 1566 cm^−1^ (2b) and 1566 cm^−1^ (2c) were in close proximity with the theoretical values of C=N stretching frequencies.

##### 3.6.9.3. C-N vibration

The C-N stretching vibrations of compound 2a, 2b and 2c were observed at the frequencies of 1207.32, 1180.35 and 1205.98 cm^−1^, respectively. The theoretically calculated values of C-N stretching of compounds 2a, 2b and 2c exhibit excellent concurrence with the experimental values 1179.9 cm^−1^ (2a), 1189.0 cm^−1^ (2b) and 1217.1 cm^−1^ (2c).

##### 3.6.9.4. C-H vibration

The stretching frequency of C-H of diazole ring was observed at 3342.85, 3345.37 and 3353.77 cm^−1^ for compound 1, 2 and 3, respectively. The symmetric and asymmetric stretching of benzene C-H appeared at 3250.02 and 3241.86 cm^−1^ for compound 1, 3250.84 and 3241.37 cm^−1^ for compound 2, and 3255.97 and 3243.68 cm^−1^ for compound 3. The symmetric C-H stretching of CH_3_ was observed in compound 1 at 3080.79 and 3148.93 cm^−1^, and in compound 2 at 3081.97, 3054.58, 3064.12 and 3051.75 cm^−1^, whereas the asymmetric C-H stretching of CH_3_ was observed in compound 1 at 3202.61 and 3138.69 cm^−1^, and in compound 2 at 3192.80, 3128.99, 3146.36 and 3139.23 cm^−1^. The CH_2_ symmetric C-H stretching was observed at 3088.41 and 3063.20 cm^−1^ (compound 1), 3045.97 cm^−1^ (compound 2) and 3103.97 and 3083.84 cm^−1^ (compound 3), while CH_2_ asymmetric C-H stretching was observed at 3155.84 and 3112.04 cm^−1^ (compound 1), 3110.90 cm^−1^ (compound 2) and 3154.06 and 3144.76 cm^−1^ (compound 3). The symmetric and asymmetric C-H stretching frequency of CH_2_ of cyclohexane in compound 2 were observed at 3077.70 and 3104.61 cm^−1^, respectively. Moreover, the frequencies of C-H bending, scissoring, rocking, wagging and twisting are given in electronic supplementary material, data (electronic supplementary material, tables S4–S6).

## 4. Conclusion and prospective

The synthesis of benzimidazolium-based analogues with variation in the alkyl group at N-1 and N-3 positions have been successfully achieved in good yields. All the compounds were characterized by the spectroscopic techniques and found stable to air and moisture both in the solid as well as in solution state. Single crystal XRD of these prepared compounds showed that cations were connected with anions and waters by H-bonding, whereas the components of same nature were not interlinked by H-bonding in compounds. Water and anion act as bridge to connect cations with each other in compounds. Crystal packing of DBIC and BMBB was further stabilized by offset π⋯π stacking interactions, whereas no π⋯π stacking interactions of significant strength was found in IMBC. Hirshfeld surface analysis inferred that H⋯H contact was the most significant contributor of the crystal packing in compounds but the contribution of H⋯C contact was larger in DBIC as compared with in BMBB and IMBC. Void analysis predicted that the compounds will have good mechanical response. The computational findings supported the experimental spectral data, especially those of UV-Vis and IR spectra. Moreover, the results revealed that the the compound 2c was more stable than other compounds. Moreover, NLO response of compound 2b was better than compounds 2a and 2c.

## Data Availability

Supplementary material is available online [90].
